# Data of the molecular dynamics simulations of mutations in the human connexin46 docking interface

**DOI:** 10.1016/j.dib.2016.01.067

**Published:** 2016-02-13

**Authors:** Patrik Schadzek, Barbara Schlingmann, Frank Schaarschmidt, Julia Lindner, Michael Koval, Alexander Heisterkamp, Anaclet Ngezahayo, Matthias Preller

**Affiliations:** a Institute of Biophysics, Leibniz University Hannover, Germany; b Division of Pulmonary, Allergy and Critical Care and Sleep Medicine, Department of Medicine and Department of Cell Biology, Emory School of Medicine, Atlanta, GA, USA; c Institute of Biostatistics, Leibniz University Hannover, Germany; d Department of Cell Biology, Emory University, Atlanta, GA, USA; e Institut für Quantenoptik, Leibniz Universität Hannover, Deutschland; f Center for System Neurosciences (ZSN), Hannover, Germany; g Institute for Biophysical Chemistry, Hannover Medical School (MHH), Hannover, Germany; h Center for Structural Systems Biology, German Electron Synchrotron (DESY), Hamburg, Germany

**Keywords:** CL, cytoplasmic loop, Cx, connexin, DOPE, discrete optimized protein energy, E2, second extracellular loop, HB, hydrogen bond, hCx, human connexin, MD, molecular dynamics, SCAM, substituted cysteine accessibility method, wt, wild type

## Abstract

The structure of hCx26 derived from the X-ray analysis was used to generate a homology model for hCx46. Interacting connexin molecules were used as starting model for the molecular dynamics (MD) simulation using NAMD and allowed us to predict the dynamic behavior of hCx46wt and the cataract related mutant hCx46N188T as well as two artificial mutants hCx46N188Q and hCx46N188D. Within the 50 ns simulation time the docked complex composed of the mutants dissociate while hCx46wt remains stable. The data indicates that one hCx46 molecule forms 5–7 hydrogen bonds (HBs) with the counterpart connexin of the opposing connexon. These HBs appear essential for a stable docking of the connexons as shown by the simulation of an entire gap junction channel and were lost for all the tested mutants.

The data described here are related to the research article entitled “The cataract related mutation N188T in human connexin46 (hCx46) revealed a critical role for residue N188 in the docking process of gap junction channels” (Schadzek et al., 2015) [1].

Specifications Table

**Table:** 

Subject area	*Computational Biology*
More specific subject area	*Molecular dynamics simulation of the connexin docking interface*
Type of data	*Figure, diagram, videos*
How data was acquired	*Homology modelling using MODELLER*[2]*and the crystal structure of hCx26 (PDB ID: 2ZW3)*[3]*; hCx46 connexins were superimposed on the hCx26 connexins chains in the connexon derived from the X-ray structure*[3]. *The molecular dynamics simulation of interacting hCx46 connexins was performed using NAMD 2.9*[4]*and the CHARMM27 force field*[5]
Data format	*Analyzed*
Experimental factors	*The interacting hCx46 connexins were placed in a cubic box filled with TIP3P water*[6]*and Na*^*+*^*-Ions; the disulfide bridges were created by patches. Temperature (310 K) and pressure (101.3 kPa) were maintained constant. The system was equilibrated for 5 ns and the dynamic was followed for 50 ns at steps of 1 fs*
Experimental features	*Simulation of connexin-connexin docking interaction*
Data source location	Institute for Biophysical Chemistry, Hannover Medical School (MHH), Hannover, Germany; Center for Structural Systems Biology, German Electron Synchrotron (DESY), Hamburg, Germany
Data accessibility	*The data is with this article*

Value of the data

• The data allow a prediction of the dynamic behavior of hCx46 molecules during docking process by molecular dynamics simulation.
• The hydrogen bonds formed by N188 of hCx46 within a connexon with residues R180, T189 and D191 of the counterpart connexin in the connexon of the adjacent cell appear to be crucial for the interacting connexin molecules to form a stable complex.
• hCx46 formed 5–7 HBs with the opposing connexins in the counterpart connexon of the adjacent cell, which stabilize the docked complex and the formation of a gap junction channel.
• Crystallization of connexins is still very difficult. However, the presented data for hCx46 shows that the structure of hCx26 derived from the X-ray analysis can be used to predict the molecular dynamic behaviors of other connexins.

## 1 Data

In this Data article we share molecular dynamics simulation data of Cx46 molecules. In our data we simulated the influence of different N188 mutation of hCx46.

## 2 Experimental design, materials and methods

### 2.1 Structural modeling and molecular dynamics simulations

To produce the data, a structural model of hCx46 was generated using MODELLER [2], and the high-resolution crystal structure of hCx26 (PDB ID: 2ZW3) [3] was taken as a template. Both connexins belong to the K/R-N group [7] and have a high sequence homology in the second extracellular loop E2, the essential region for connexon docking. Therefore the usage of hCx26 as template for this region seems to be acceptable. The MODELLER objective function and the discrete optimized protein energy (DOPE) were used for the evaluation and selection of the models. The CL loop conformation, which is missing in the template structure, was subsequently optimized. The gap junction channel of hCx46 was constructed by superposition of the hCx46 connexins on the hCx26 connexin chains in the connexon derived from the X-ray structure [3]. A two-fold symmetry operation was used to build the second hemichannel. A pair of interacting hCx46 molecules of opposing hemichannels was selected as the model system for molecular dynamics simulations, and for the different mutants in which the aspargine residue at position 188 was replaced by either threonine, glutamine or aspartic acid. The four simulations – hCx46wt, hCx46N188T, hCx46N188Q and hCx46N188D – were immersed individually in a cubic box filled with explicit TIP3P water molecules [6], and the net charge was neutralized by adding Na^+^ counter ions. To keep disulfide bridges stable, patches were implemented between residues C54 - C192, C61 - C186, and C65 - C181 of hCx46, which correlate with the disulfide bridges C53 - C180, C60 - C174, and C64 - C169 in hCx26. All molecular dynamics simulations were conducted using NAMD 2.9 [4] and the CHARMM27 force field [5]. A 12 Å cutoff was used for non-bonded short-range interactions, and long-range electrostatics were treated with the particle-mesh Ewald method [8]. Temperature and pressure were maintained at 310 K and 101.3 kPa using Langevin dynamics and the Langevin piston method. The simulation time step was 1 fs. Each simulation system was first energy minimized and subsequently equilibrated for approximately 5 ns – during which the systems converged towards a backbone RMSD of 5–7 Å - prior to production runs. Molecular dynamics (MD) simulations were conducted for 50 ns each at the Computer Cluster of the Norddeutscher Verbund für Hoch- und Höchstleistungsrechnen (HLRN). Simulations of the entire hCx46wt and hCx46N188Q gap junction channels were prepared and treated analogously to the above described procedure.

### 2.2 Molecular dynamics simulation of interacting hCx46 connexins

**Fig. 1:**
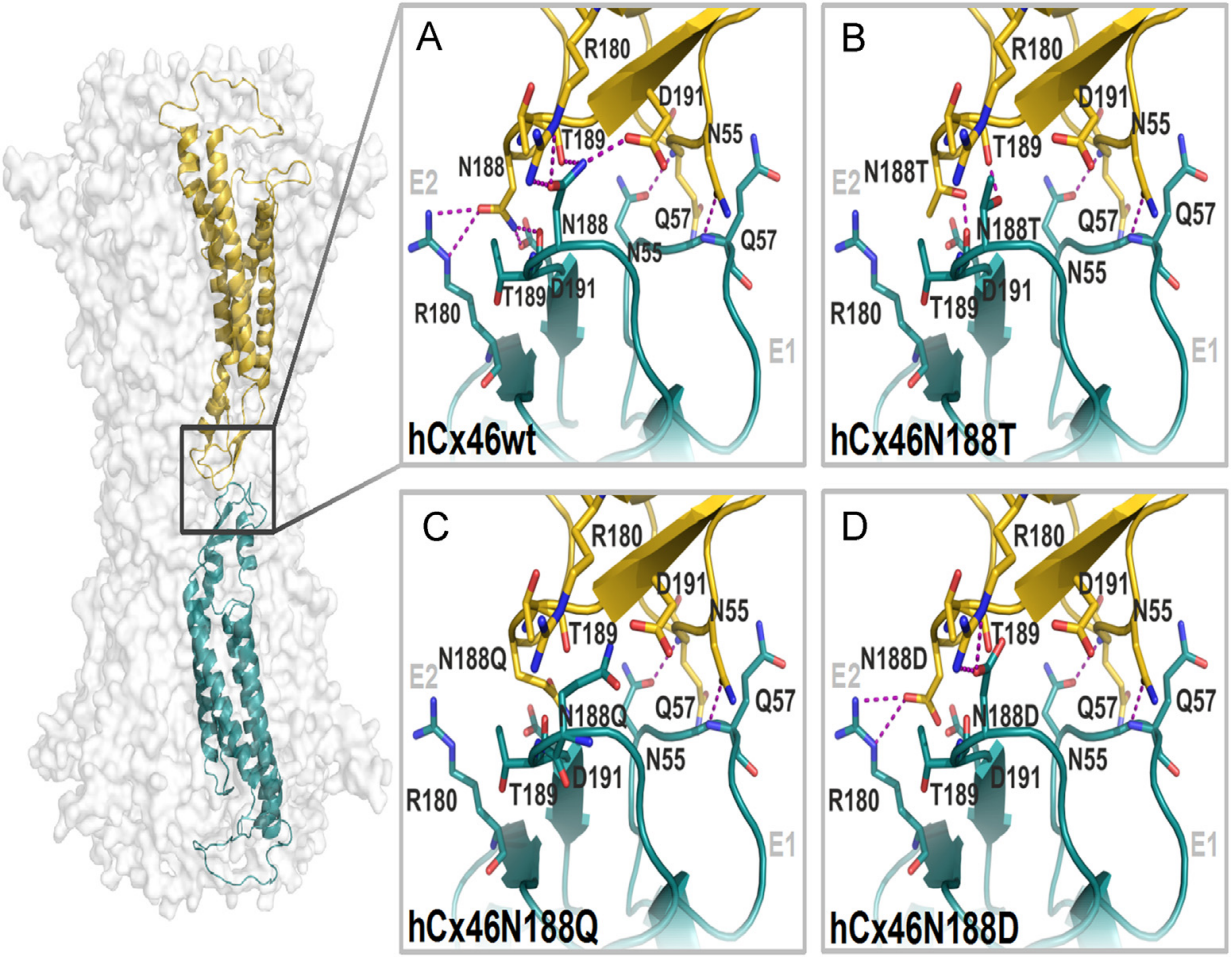
Binding interface and HBs network of the wild type (A) and the three mutants, hCx46N188T (B), hCx46N188Q (C) and hCx46N188D (D). The three N188 mutations disturb the HB network to varying degrees.

**Fig. 2:**
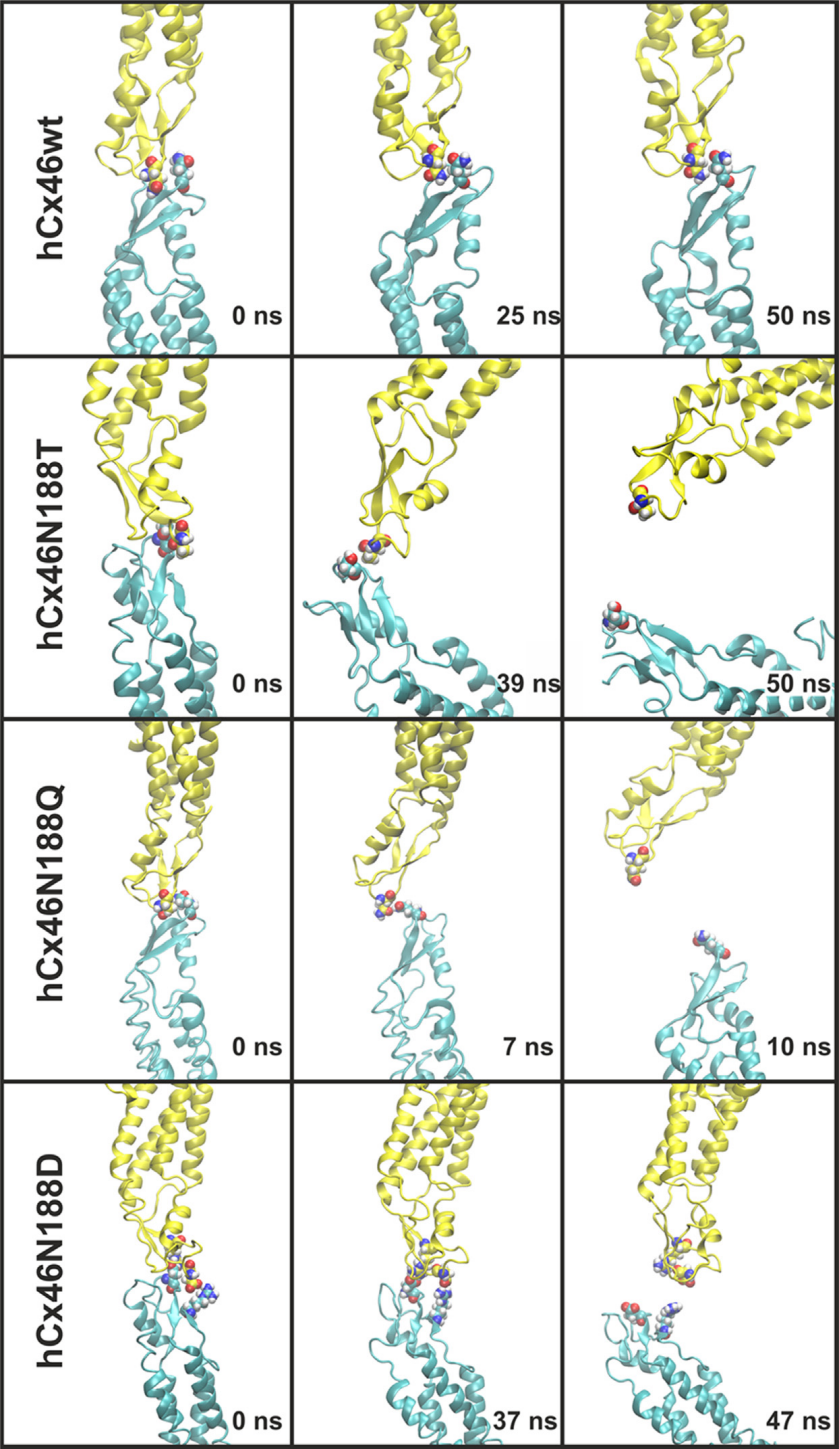
Snapshots from the molecular dynamics simulations of hCx46wt and the three mutants.

**Fig. 3:**
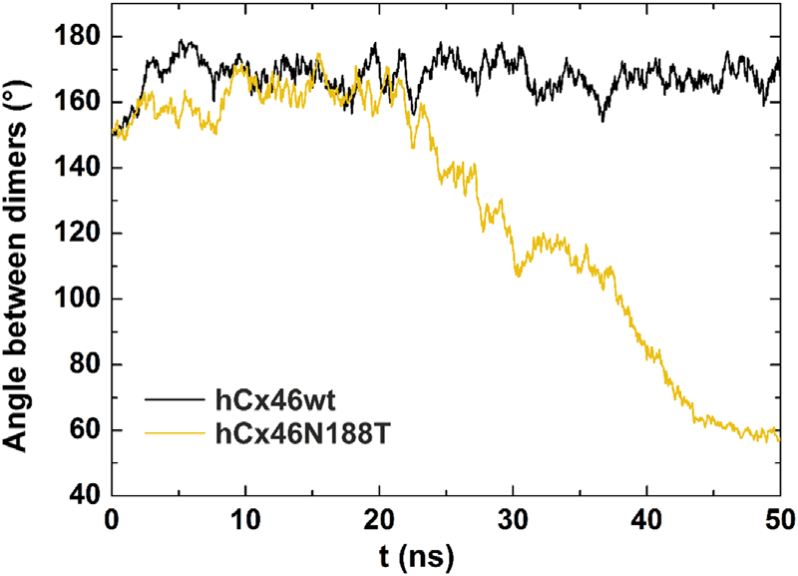
Diagram depicting the angle between the interacting connexins along the simulation time for hCx46wt (black) and hCx46N188T (gold). While the N188T mutation leads to a kinking of the connexins relative to each other and finally to the dissociation of the complex, hCx46wt connexins remain rather constant with an angle around 170°.

The data shows the influence of the N188T mutation compared to the wild type, molecular dynamics simulations were performed over 50 ns simulation time. The artificial mutation N188Q reintroduces a carboxamide group at position 188 but is bulkier. In the second artificial mutation N188D, the asparagine residue is replaced by the equally sized aspartic acid residue. Fig. 1 shows the data of the HB network surrounding residues N188, T188, Q188 and D188, [1] which got disrupted in all the mutants, leading to a destabilization of the complex during the MD simulation (Fig. 2, movies of the molecular dynamics simulation: supplementary material S1–S4). Furthermore, monitoring the angles between the interacting connexins along the simulation time for the wild type and the N188T mutant showed a stable angle of about 170° between the hCx46wt monomers, while the hCx46N188T monomers bent relative to each other, reducing the angle to about 60° during the trajectory (Fig. 3).

Supplementary material related to this article can be found online at doi:10.1016/j.dib.2016.01.067.

The following is the Supplementary material related to this article Movie S1, Movie S2, Movie S3, Movie S4.

Movie S1 Supplementary video 1. Movie of the MD trajectory showing a close-up view of the binding interface between two **hCx46wt** connexins of opposing connexons. The complex was stable over the entire simulation.

Movie S2 Supplementary video 2. Movie of the MD trajectory showing a close-up view of the binding interface between two **hCx46N188T** connexins of opposing connexons. The complex was not stable.

Movie S3 Supplementary video 3. Movie of the MD trajectory showing a close-up view of the binding interface between two **hCx46N188Q** connexins of opposing connexons. The complex dissociated within the first 10 ns of simulation.

Movie S4 Supplementary video 4. Movie of the MD trajectory showing a close-up view of the binding interface between two **hCx46N188D** connexins of opposing connexons. The complex was not stable.

### 2.3 N188D introduces a negative charge

**Fig. 4:**
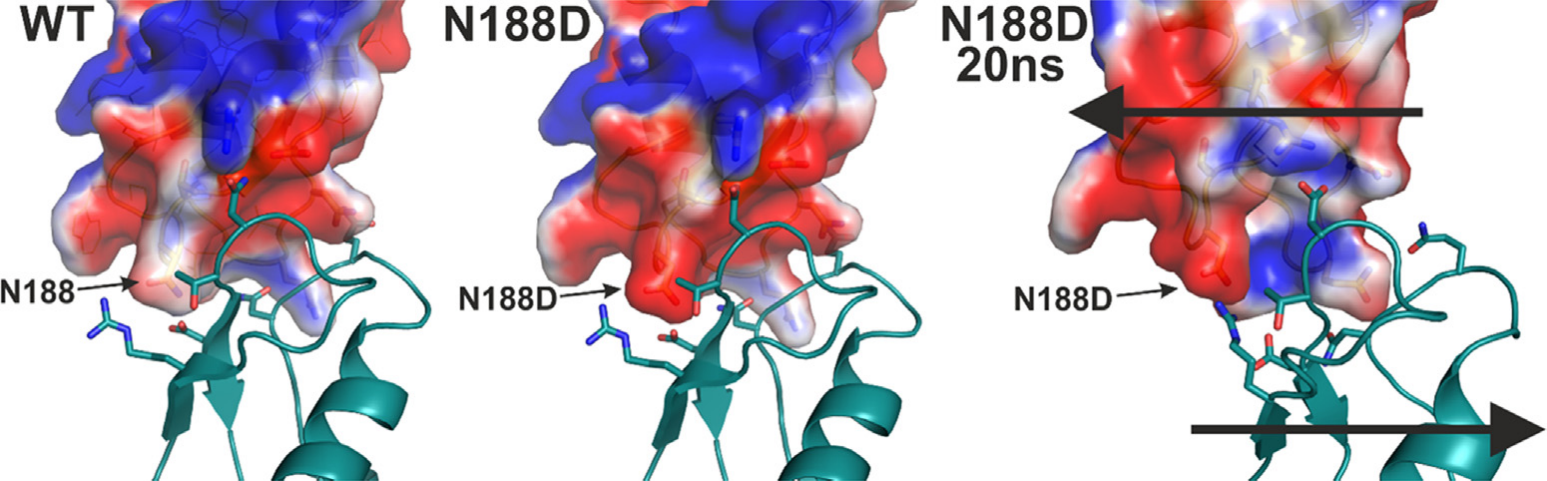
The snapshot sequence shows the electrostatic repulsion of the hCx46N188D mutation with D191 (long arrows). Blue areas symbolize a positive charge, white a neutral charge and the red colored area is negatively charged.

The data of the artificial mutation N188D introduces a negative charge that seems to cause an electrostatic repulsion. Fig. 4 shows the electrostatic surface potential of the molecules of wild type connexin compared to the N188D mutation (blue areas symbolize positive charges, white areas indicate neutral regions and red colored areas represent negatively charged regions) as well as the repulsion after 20 ns simulation time (long arrows).

Furthermore, we tested the effect of reciprocal charge mutations N188D and D191N on our Cx46 model system during MD simulations (see supplementary video S5) since our data shows that the residue N188 forms a hydrogen bond with D191. Despite the reported rescue function of such reciprocal charge mutations for the Cx32/Cx26 complex [4], [9], the Cx46 complex dissociated within 50 ns in our simulations.

Supplementary material related to this article can be found online at doi:10.1016/j.dib.2016.01.067.

The following is the Supplementary material related to this article Movie S5.

Movie S5 Supplementary video 5.Movie of the MD trajectory showing a close-up view of the binding interface between **hCx46N188D and hCx46D191N** connexins of opposing connexons. The reciprocal charge mutations N188D and D191N on Cx46 did not stabilize the complex, despite the reported rescue function of such reciprocal charge mutations for the Cx32/Cx26 complex (Gong et al. [9]), the Cx46 complex dissociated within 50 ns simulations time in our model.

### 2.4 Data from a model of whole gap junction channels

**Fig. 5:**
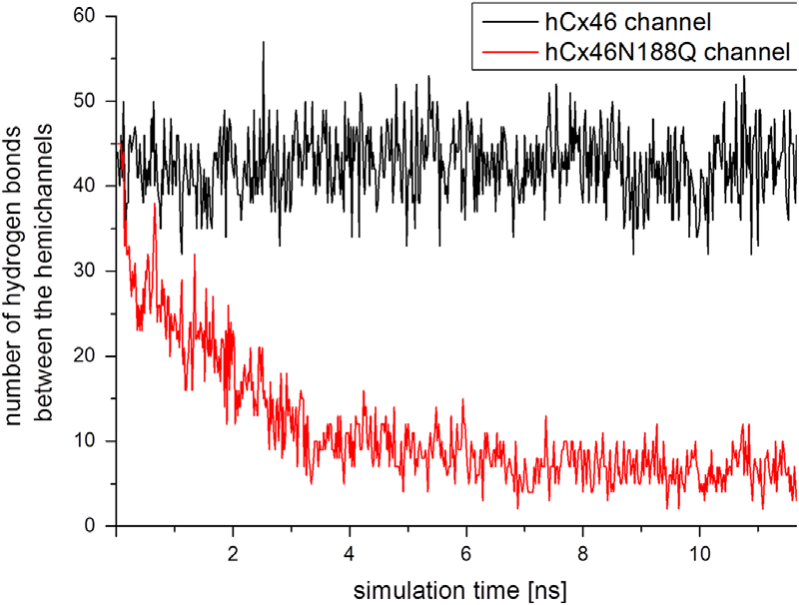
The number of HBs between the hemichannels composed of hCx46wt (black) and hCx46N188Q (red). According to Gong et al. [9], the stability of a gap junction channel is achieved if an average of 18 HBs is achieved. For the hCx46N188Q channel the number of HBs is reduced to less than 10 within the first 3 ns leading to the dissociation of the complex. The supplemental movie S7 shows the dissociation. In comparison, movie S6 shows that hCx46wt hemichannels form a stable complex.

Simulations were performed for the entire hCx46wt and hCx46N188Q gap junction channels (see movies in the supplementary material S6 and S7). The data shows that hCx46wt connexons interact through an average of 40 HBs in the simulations. These HBs stabilize the whole gap junction channel complex along the trajectory (movie S6). For the hCx46N188Q connexins, the connexons formed also about 40 HBs at the beginning of the simulation. However, the number of HBs decreased rapidly within the first 3–4 ns of simulation and the channel complex started to dissociate (Fig. 5 and movie S7). Nevertheless, after 10 ns complete dissociation of the channel was not achieved and an average of 5 HBs were still detectable.

Supplementary material related to this article can be found online at doi:10.1016/j.dib.2016.01.067.

The following is the Supplementary material related to this article Movie S6, Movie S7.

Movie S6 Supplementary video 6. Movie of the MD trajectory showing a close-up view of the binding interface between two **hCx46wt opposing entire connexons**. The complex was stable over the entire simulation.

Movie S7 Supplementary video 7. Movie of the MD trajectory showing a close-up view of the binding interface between two **hCx46N188Q opposing entire connexons**. The complex dissociated within 10 ns of the simulation.
